# Restoration of hair follicle inductive properties by depletion of senescent cells

**DOI:** 10.1111/acel.14353

**Published:** 2024-11-29

**Authors:** Alberto Pappalardo, Jin Yong Kim, Hasan Erbil Abaci, Angela M. Christiano

**Affiliations:** ^1^ Department of Dermatology Columbia University New York New York USA; ^2^ Department of Genetics and Development Columbia University New York New York USA

**Keywords:** cellular senescence, dasatinib, dermal papilla, hair follicle, quercetin, regeneration, senescence‐associated secretory phenotype, senolytic

## Abstract

Senescent cells secrete a senescence‐associated secretory phenotype (SASP), which can induce senescence in neighboring cells. Human dermal papilla (DP) cells lose their original hair inductive properties when expanded *in vitro*, and rapidly accumulate senescent cells in culture. Protein and RNA‐seq analysis revealed an accumulation of DP‐specific SASP factors including IL‐6, IL‐8, MCP‐1, and TIMP‐2. We found that combined senolytic treatment of dasatinib and quercetin depleted senescent cells, and reversed SASP accumulation and SASP‐mediated repressive interactions in human DP culture, resulting in an increased Wnt‐active cell population. In hair reconstitution assays, senolytic‐depleted DP cells exhibited restored hair inductive properties by regenerating de novo hair follicles (HFs) compared to untreated DP cells. In 3D skin constructs, senolytic‐depleted DP cells enhanced inductive potential and hair lineage specific differentiation of keratinocytes. These data revealed that senolytic treatment of cultured human DP cells markedly increased their inductive potency in HF regeneration, providing a new rationale for clinical applications of senolytic treatment in combination with cell‐based therapies.

## Graphical abstract

Human dermal papilla cells (DP) lose hair inductivity and rapidly become senescent when expanded in vitro. We found a DP‐specific SASP including IL‐6, IL‐8, MCP‐1, and TIMP‐2. Senolytic treatment with dasatinib and quercetin depleted senescent DP and restored hair inductivity in hair reconstitution assays in mice, and induced hair lineage keratinocytes differentiation in engineered skin. These findings provide a new rationale for clinical applications of senolytic treatment in combination with cell‐based therapies.

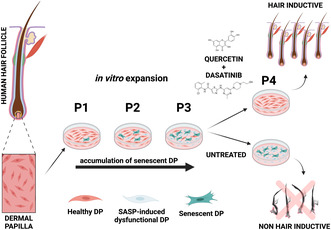

**Abbreviations**
3D: three‐dimensional
CCL2: chemokine (C‐C motif) ligand 2 (also known as MCP‐1)
DAPI: 4′,6‐diamidino‐2‐phenylindole
DF: dermal fibroblast
DMEM: Dulbecco's Modified Eagle Medium
DP: dermal papilla
ECM: extracellular matrix
FBS: fetal bovine serum
GEO: gene expression omnibus
HF: hair follicle
IL‐6: interleukin 6
IL‐8: interleukin 8
MCP‐1: monocyte chemoattractant protein‐1 (also known as CCL2)
P1, P2, P3, etc.: Passage 1, Passage 2, Passage 3, etc. (related to cell culture)
PBS: phosphate‐buffered saline
p‐Stat3: phosphorylated signal transducer and activator of transcription 3
RNA‐seq: RNA sequencing
SASP: senescence‐associated secretory phenotype
SASP: senescence‐associated secretory phenotype
SA‐β‐Gal: senescence‐associated β‐galactosidase
SCID: severe combined immunodeficiency
SHO: severe combined immunodeficiency hairless outbred
SSP1: secreted phosphoprotein 1 (also known as Osteopontin)
TGF‐β: transforming growth factor beta
TIMP‐2: tissue inhibitor of metalloproteinases‐2
UV: ultraviolet

## INTRODUCTION

Cellular senescence was originally defined as finite proliferative capacity of cells in *in vitro* culture, after which human fibroblasts entered a metabolically active, but irreversibly arrested proliferative state (Sharpless & Sherr, 2015). Under physiological conditions such as tissue homeostasis or development, senescence can be a beneficial cell state for regenerative tissue remodeling through the secretion of a group of cytokines and chemokines, known as the senescence‐associated secretory phenotype (SASP) (Sharpless & Sherr, 2015). SASP factors create a local inflammatory environment and facilitate the recruitment of immune cells, thereby promoting the immune‐mediated clearance of senescent cells from the tissue microenvironment (Munoz‐Espin & Serrano, 2014; Ritschka et al., 2017). However, if senescent cells are not efficiently cleared, the accumulation of the SASP leads to pathologic dysfunction of nearby non‐senescent cells, including tissue stem cells, which then contribute age‐related degenerative phenotypes (Munoz‐Espin & Serrano, 2014).

Many approaches have been attempted for human organ regeneration from adult cells, but these have been largely unsuccessful without introducing cell fate modifications such as reprogramming into induced pluripotent stem cells (Abaci et al., 2017; Gledhill et al., 2015; Itoh et al., 2011; Umegaki‐Arao et al., 2014). In the hair follicle (HF), research has focused on utilizing the regenerative properties of human dermal papilla (DP) cells, which are the inductive mesenchymal cell population of the HF, to direct human HF neogenesis (Leiros et al., 2014; Lim et al., 2013; Qiao et al., 2009) (clinical trial NCT01451021). However, despite a strong translational interest, subsequent efforts to elicit human HF neogenesis with human DP cells after a significant expansion in *in vitro* culture have been largely unsuccessful. Paradoxically, when intact DP are removed from the HF and expanded in culture, they lose contextual and positional cues and their inductive potency declines rapidly (Higgins et al., 2010, 2013; Jahoda et al., 1993; Kishimoto et al., 1999, 2000; McElwee et al., 2003; Ohyama et al., 2012; Reynolds & Jahoda, 1996).

Unlike cultured rodent DP, which readily direct HF induction, human DP do not reacquire hair inductivity in late passages (>passage 4), and the approaches described in literature, such as induction of WNT/β‐catenin pathway (Shimizu & Morgan, 2004; Zhou et al., 2016), chemicals (Kim et al., 2006), 3D culture conditions (Higgins et al., 2010, 2013; Qiao et al., 2009), hormones (Kageyama et al., 2023), extracellular matrix (ECM) (Liu et al., 2023), transfection with plasmids encoding transcription factors (Abaci et al., 2018), and conditioned medium (Abreu et al., 2021), have proven effective only on early passage human DP cells.

It is not understood why human DP cells lose their inductive properties in culture, or how to fully restore their original inductivity after significant in vitro expansion for human HF regeneration. In light of the current evidence, it is likely that 2D culture of human DP disrupts mechanosignaling and alters the phenotype of these cells. Using chromatin accessibility profiling, it was recently demonstrated that human DPs are enriched for motifs of the TEAD family of transcription factors (which associates with YAP/TAZ in the nucleus) and are capable of bone differentiation upon mechanical (and chemical) stimulation, unlike papillary fibroblasts (Logan et al., 2024). This could explain why when DP are cultured as cell aggregates, with reestablishment of a physiological cell–cell and cell‐ECM contact, the DP gene signature is partially restored (Higgins et al., 2010, 2013).

Recently, it was reported that the DP exhibits senescent‐like characteristics and expresses the SASP in aged mice (Shin et al., 2020). Cellular senescence in the DP is associated with progressive dysfunction within the mesenchymal stem cell pool of the HF (Shin et al., 2020). The inability to clear senescent stem cells from tissues has emerged as a direct contributor to aging and tissue degeneration in many tissues (Di Micco et al., 2020). Furthermore, under non‐physiologic conditions such as *in vitro* culture, where senescent cells are never cleared, the persistence of senescent cells may result in a bystander effect on nearby non‐senescent cells, thus hampering the utilization of human adult stem cells for organ regeneration. Since senescent cells have pleiotropic effects on many tissues both locally and systemically (Kirkland et al., 2017), a novel class of drugs, known as senolytics, has been developed to selectively deplete senescent cells from tissues (Kirkland et al., 2017). Interestingly, it was previously shown that senolytic treatment could partially reverse age‐related hair loss in both normal aged mice, as well as genetically fast‐aging mice (Baar et al., 2017). These findings suggested that depletion of senescent cells can restore tissue homeostasis within the HF and promote hair growth in aged mice (Baar et al., 2017), inviting further investigation into the effects of senescent cells on hair growth.

**FIGURE 1:**
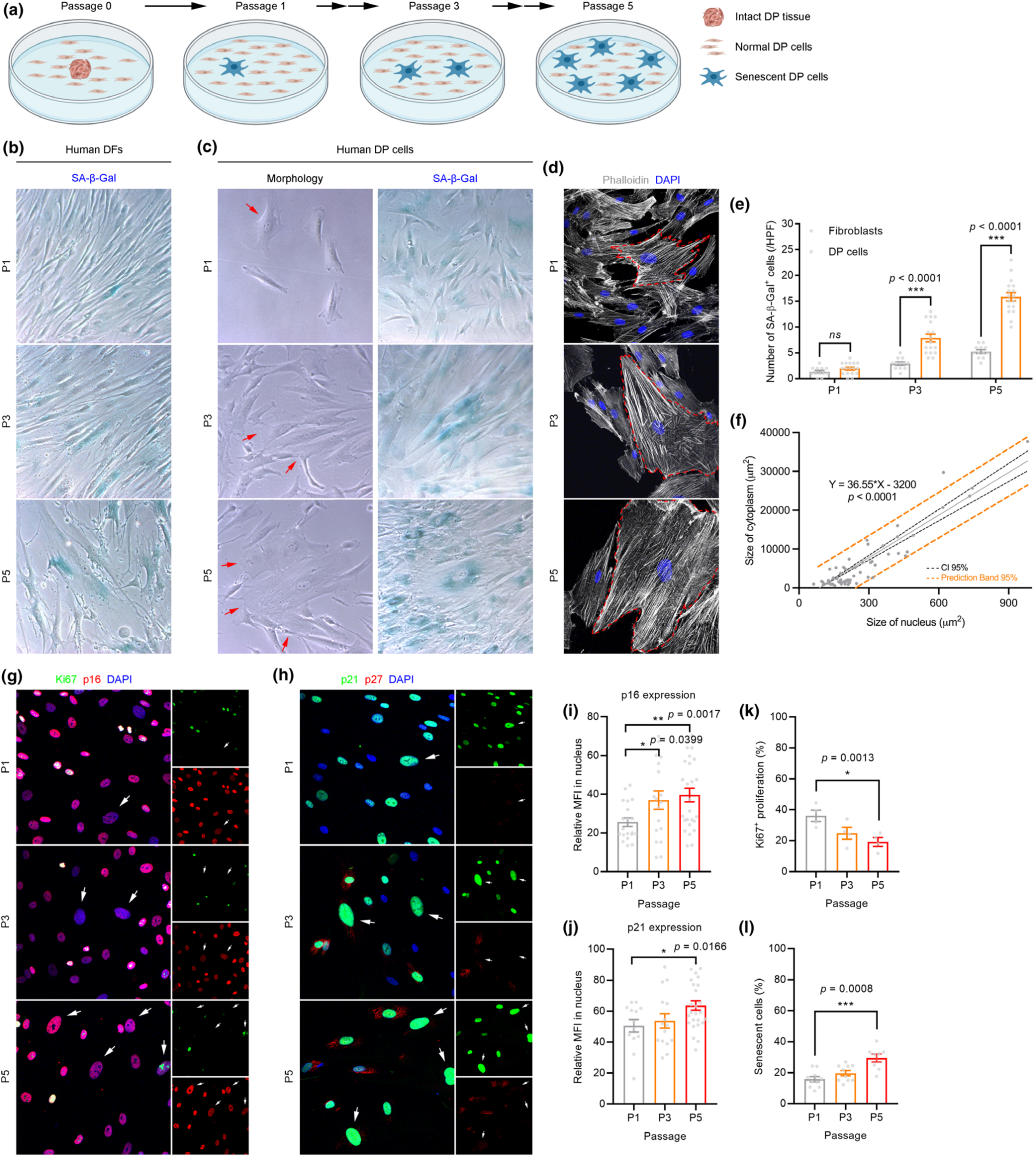
Senescent DP cells emerge as early as passage 1 in human DP culture. (a) Experimental scheme to characterize the cellular senescence of human DP cells after *in vitro* expansion. (b) Cell morphology and SA‐β‐Gal activity in human DF culture showing that SA‐β‐Gal^+^ senescent DF cells were increased over passage. (c) Cell morphology and SA‐β‐Gal activity in human DP culture showing that SA‐β‐Gal^+^ senescent DP cells were increased over passage. (d, f) Immunofluorescence and quantification of human DP culture (*n* = 64 cells from 3 biological replicates) showing that senescent DP cells exhibited markedly expanded cytoplasm and enlarged nuclear size showing a strong positive correlation between the size of cytoplasm and the size of nucleus. (e) Quantification of number of SA‐β‐Gal^+^ senescent cells in human DF and DP cultures (*n* = 6 DF replicates and 8–9 DP replicates from 2 independent donors) showing that SA‐β‐Gal^+^ cells senescent were more prevalent in DP culture than in DF culture as early as P1 to P5. (g, i) Immunofluorescence and quantification of senescent DP cells (*n* = 114 [P1], 99 [P3], 81 [P5] cells from 2 independent donors) showing that nuclear p16 expression was increased in senescent DP cells over subsequent passage. (h, j) Immunofluorescence and quantification of senescent DP cells (*n* = 129 [P1], 107 [P3], 101 [P5] cells from 2 independent donors) showing that nuclear p21 expression was increased in senescent DP cells over subsequent passage and that p21^+^ cells showed no p27 expression in the nucleus, which indicates senescence, not quiescence. (k) Quantification of Ki67^+^ cells showing that the percentage of proliferating cells decreased over subsequent passages (*n* = 4 biological replicates/group). (l) Quantification of senescent cells showing that the percentage of proliferating cells decreased over subsequent passages (*n* = 8 biological replicates/group). Data are presented as the mean ± s.e.m. *p < 0.05, **p < 0.01, ***p < 0.001 (two‐way analysis of variance with Sidak's multiple comparisons test) in (e); (linear regression test) in (f); (Welch's *t* test) in (i–l).

To characterize the emergence of cellular senescence in human DP cells under *in vitro* culture, we isolated human DPs from HFs, cultured them for several passages, and compared them to human dermal fibroblasts (DF) isolated from the scalp of same donor (Figure 1a). We selected DF as a control since they share a common progenitor with DPs (Driskell et al., 2013), are non‐hair inductive, are isolated from the dermis surrounding the HFs, which slowly accumulate senescent cells during passage (Raffetto et al., 1999), and are extensively studied in the field of senescence (Contrepois et al., 2017; Coppe et al., 2008; Idda et al., 2020; Jo et al., 2020; Kong et al., 2023; Rube et al., 2021; Waldera Lupa et al., 2015; Wang et al., 2009). To detect senescent cells, we examined senescence‐associated‐β‐galactosidase (SA‐β‐Gal) activity from passage 1 (P1) to passage 5 (P5) and found SA‐β‐Gal^+^ cells both in human DF and DP cultures (Figure 1b). SA‐β‐Gal^+^ cells were more prevalent in DP culture than in DF culture as early as P1, and exhibited a flattened and enlarged morphology (Figure 1c,e). Senescent DP cells showed a markedly expanded cytoplasm and enlarged nuclear size with a strong positive correlation between the size of cytoplasm and the size of nucleus (Figure 1d,f). No proliferation was observed in senescent DP cells as determined by Ki67 expression (Figure 1g). By quantification of protein expression levels and nuclear size, we found that nuclear p16 expression (Figure 1g,i) and p21 expression (Figure 1h,j) was increased in senescent DP cells over subsequent passages, and that p21^+^ senescent DP cells showed no p27 expression in the nucleus (Figure 1h), which indicate the senescent state, and not quiescence (Perucca et al., 2009). Over subsequent passages of DP cells in culture, the percentage of proliferating cells decreased (Figure 1k) whereas the percentage of senescent cells increased (Figure 1l). Taken together, we observed that cellular senescence occurred rapidly in human DP cultures, as early as one passage after explant, and further that these senescent DP cells become the predominant cell population, leading to cellular heterogeneity.

**FIGURE 2:**
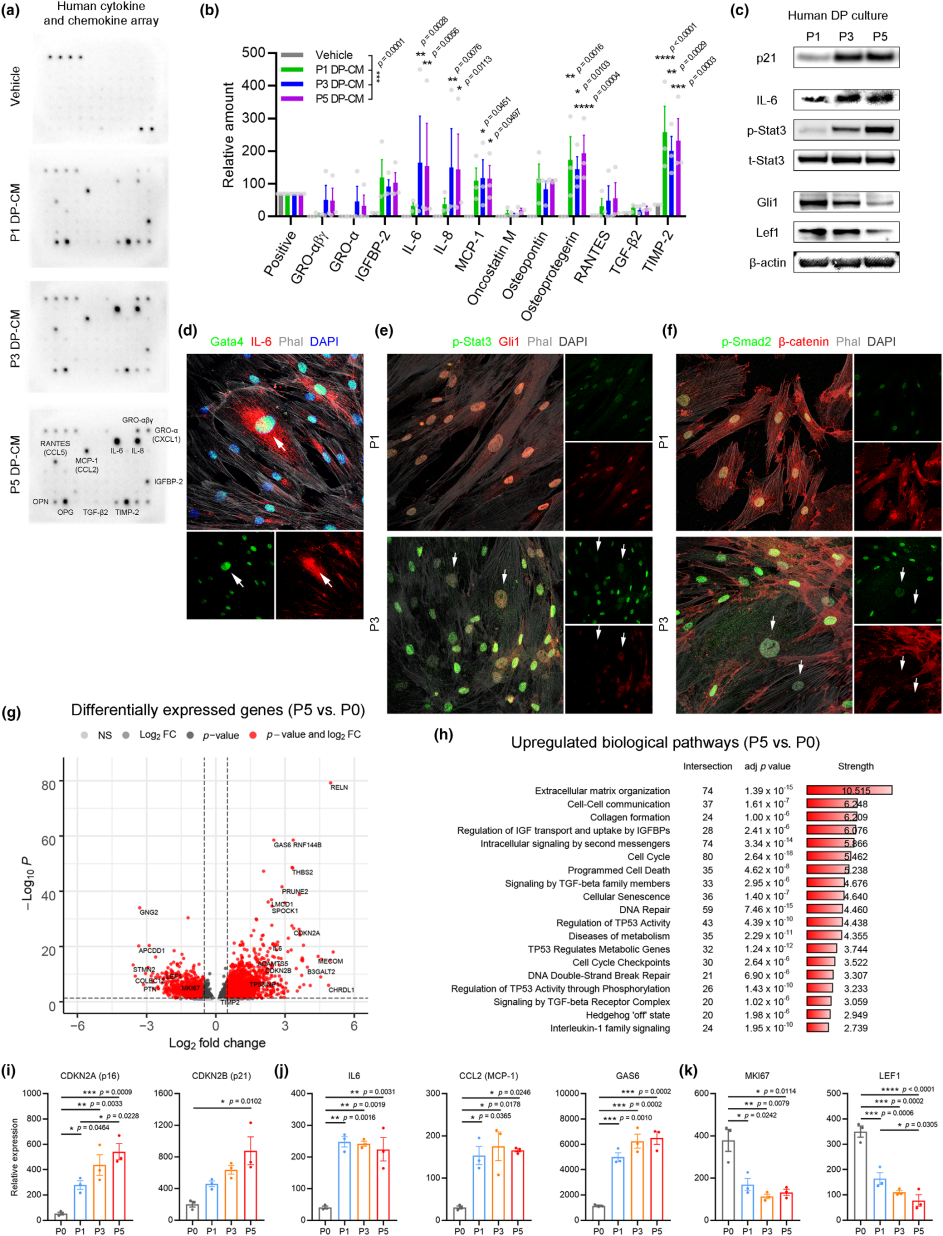
SASP from senescent DP cells have repressive effects on nearby non‐senescent DP cells. (a, b) Protein arrays and quantification of the conditioned medium of human DP culture (*n* = 3 biological replicates) showing that a unique combination of SASP factors, including IL‐6, IL‐8, MCP‐1, TIMP‐2, osteopontin, and osteoprotegerin, accumulated in the conditioned medium over passage. (c) Western blot of cultured DP cells showing that marked increase of p21 and p16 with IL‐6 and p‐Stat3 in P5 DP culture compared to P1 DP culture in contrast to marked decrease of Gli1 and Lef1 in P5 DP culture compared to P1 DP culture. (d) Immunofluorescence showing that enlarged senescent cells exhibited cytoplasmic IL‐6 expression with nuclear GATA4 expression. (e, f) Immunofluorescence showing that p‐Stat3 activation (IL‐6 signaling) and p‐Smad2 activation (TGF‐β signaling) with decreased Gli1 and nuclear β‐catenin expression in non‐senescent DP cells nearby enlarged senescent DP cells in P3 DP culture compared to P1 DP culture. (g) Volcano plot of differentially expressed genes showing that RELN, GAS6, THBS2, CDKN2A, IL6, CDKN2B, and TP53INP1 were upregulated and GNG2, APCDD1, STMN2, LEF1, MKI67 were downregulated in cultured DP cells over passage (*n* = 3 biological replicates). (h) Gene ontology analysis showing that biological pathways including cellular senescence, cell–cell communication, intracellular signaling by second messengers, signaling by TGF‐beta family members, DNA repair, regulation of TP53 activity, DNA double‐strand break repair, and interleukin‐1 family signaling were upregulated in cultured DP cells. (i–k) Representative gene expression showing that the senescence drivers CDKN2A and CDKN2B were significantly upregulated along with SASP factors IL6 and CCL2, whereas MKI67 and LEF1 were significantly downregulated over passage in human DP culture (*n* = 3 biological replicates). Data are presented as the mean ± s.e.m. *p < 0.05, **p < 0.01, ***p < 0.001, ****p < 0.0001 (two‐way analysis of variance with Dunnett's multiple comparisons test) in (b); (repeated measure one‐way analysis of variance with Sidak's multiple comparisons test) in (i–k).

Senescent cells are metabolically active and produce a secretome of cytokines and chemokines, which are referred as the SASP (Di Micco et al., 2020). To identify the SASP factors that were specific to senescent DP cells, we evaluated cytokines and chemokines from the conditioned medium of cultured human DP cells over passage using protein microarrays (Figure 2a; RayBiotech AAH‐CYT‐5). We identified a unique combination of SASP factors of senescent DP cells that accumulated in the conditioned medium over passage. We found that the DP‐specific SASP factors included IL‐6, IL‐8, MCP‐1 (CCL2), TIMP‐2, osteopontin (SSP1), and osteoprotegerin (TNFRSF11B) (Figure 2b). Protein analysis of human DP culture showed a marked increase of p21 and p16 with IL‐6 and phosphorylated (p‐) Stat3 in P5 DP culture compared to P1 DP culture, as well as a marked decline of Gli1 and Lef1 expression in P5 DP culture compared to P1 DP culture (Figure 2c). To determine the source of IL‐6 in human DP culture, we stained IL‐6 and GATA4, a transcription factor associated with SASP production (Di Micco et al., 2020), and found cytoplasmic IL‐6 expression with nuclear GATA4 expression in enlarged senescent DP cells (Figure 2d). To define SASP‐mediated interactions between senescent DP cells and nearby non‐senescent DP cells, we examined p‐Stat3 (readout of IL‐6 signaling) with Gli1, and p‐Smad2 (readout of TGF‐β signaling) with β‐catenin. We found that IL‐6 signaling activation and TGF‐β signaling activation correlated with decreased Gli1 and nuclear β‐catenin expression (Figure 2e,f) in non‐senescent DP cells in P3 culture compared to P1 culture. These data suggest that senescent DP cells exert repressive effects on neighboring non‐senescent DP cells through SASP‐mediated interactions in human DP culture.

To interrogate the senescence‐associated changes in human DP cells after expansion, we analyzed global gene expression profiling using microarray analysis of cultured DP cells at P0, P1, P3, and P5 (GSE44765). Based on differential gene expression analysis (adjusted *p* < 0.05), 3738 genes (16%) were upregulated, while 1444 genes (6.3%) were downregulated over passage (Figure S1). For example, RELN, GAS6, THBS2, CDKN2A, IL6, CDKN2B, and TP53INP1 were upregulated, and GNG2, APCDD1, STMN2, LEF1, MKI67 were downregulated during passaging (Figure 2g). We identified the major biological pathways activated in cultured DP cells using the Reactome database (Figure 2h). Biological pathways that were upregulated in human DP cells after expansion included cellular senescence, cell–cell communication, intracellular signaling by second messengers, signaling by TGF‐beta family members, DNA repair, regulation of TP53 activity, DNA double‐strand break repair, and interleukin‐1 family signaling. The expression of the senescence drivers CDKN2A and CDKN2B (Figure 2i) was significantly upregulated, along with SASP factors IL6 and CCL2 (Figure 2j), whereas the expression of MKI67 and LEF1 (Figure 2k) was significantly downregulated over passage in human DP culture. Taken together, this transcriptomic profile revealed that the major changes in human DP cells after expansion in culture were senescence‐ and SASP‐associated interactions within cultured DP cells.

**FIGURE 3:**
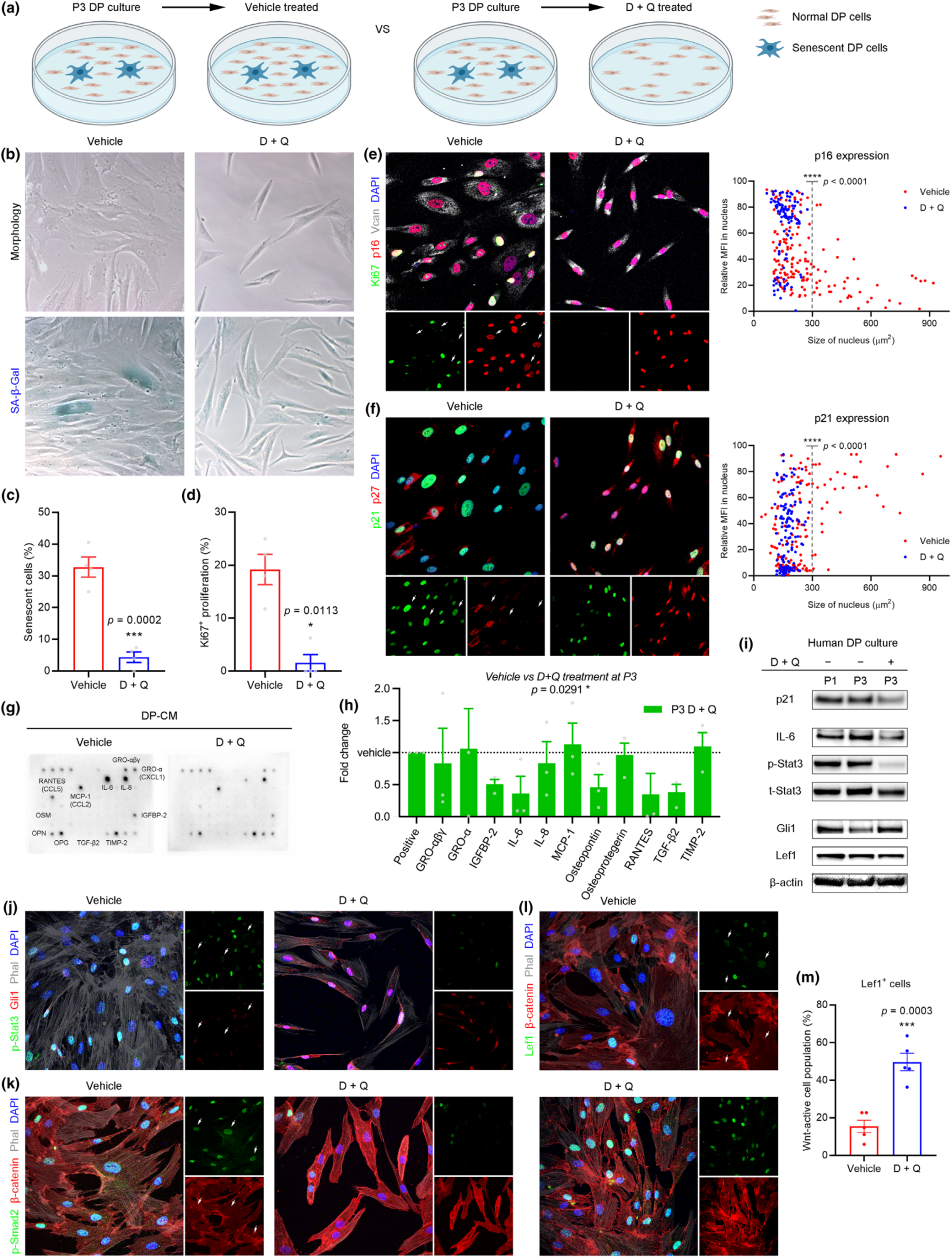
Senolytic treatment reverses senescence‐driven repressive effects in human DP culture. (a) Experimental scheme to determine the effect of senolytic treatment on human DP culture. (b) Cell morphology and SA‐β‐Gal activity of P3 DP culture after vehicle or dasatinib plus quercetin (D + Q) treatment. (c) Quantification of senescent cells (*n* = 4 biological replicates/group) showing that the percentage of senescent DP cells decreased significantly after senolytic treatment. (d) Quantification of Ki67^+^ cells (*n* = 4 biological replicates/group) showing that almost no proliferation was observed in the remaining non‐senescent DP cells after senolytic treatment. (e, f) Immunofluorescence and quantification of human DP culture after vehicle or senolytic treatment (*n* = 159 [vehicle] 109 [D + Q] cells in [e] and 148 [vehicle] 141 [D + Q] cells in [f] from 2 independent donors) showing remaining DP cells showed high p16, p21, and p27 expression in the nucleus, indicating that remaining DP cells at the end of senolytic treatment were in quiescence status in contrast to irreversible proliferation arrest in senescent DP cells. (g, h) Protein arrays and quantification of the conditioned medium of human DP culture after vehicle (dotted baseline) or senolytic treatment (columns) showing that the DP‐specific SASP factors were overall significantly reduced in the conditioned medium after senolytic treatment (two‐way ANOVA with Šídák's multiple comparisons test). (i) Western blot of human DP culture after vehicle or senolytic treatment at P3 showing that marked decrease of p21 with IL‐6 and p‐Stat3 in senolytic‐depleted P3 DP culture compared to control P3 DP culture as well as sustained expression of Gli1 and Lef1 in senolytic‐depleted P3 DP culture compared to control P3 DP culture. (j, k) Immunofluorescence showing that activation of IL‐6 signaling and TGF‐β signaling in non‐senescent DP cells was reversed after senolytic treatment, with recovered Gli1 and nuclear β‐catenin expression. (l, m) Immunofluorescence and quantification showing that the percentage of Lef1^+^ cells in cultured DP cells significantly increased in senolytic‐depleted P3 DP culture compared to control P3 DP culture (*n* = 5 biological replicates/group). Data are presented as the mean ± s.e.m. *p < 0.05, ***p < 0.001, ****p < 0.0001 (Welch's *t* test) in (c, d); (unpaired *t* test) in (e, f, m); (two‐way analysis of variance) in (h).

Senolytics are a novel class of drugs that selectively remove senescent cells to prevent negative effects within heterogeneous tissues (Di Micco et al., 2020). To identify the most potent drug(s) for eliminating senescent DP cells, we tested human DP cultures with ABT‐263 (Navitoclax), ABT‐737, ABT‐199 (Venetoclax), and dasatinib plus quercetin (D + Q) (Figure S2). We found that D + Q was the most potent senolytic combination for depleting senescent cells from human DP cultures. We treated cultured DP cells with D + Q (1+ 20 μM for 48 h) at P3 to determine whether the senescence‐driven repressive changes can be reversed by depletion of senescent cells (Figure 3a). Most senescent DP cells were depleted after 48 h of senolytic treatment, as shown by loss of flattened and enlarged cells as well as loss of SA‐β‐Gal activity (Figure 3b). The percentage of senescent cells in the remaining cell population decreased significantly after senolytic treatment (Figure 3c), and almost no proliferation was observed in the remaining non‐senescent DP cells immediately after senolytic treatment (Figure 3d). Indeed, the DP cells that remained after senolytic treatment were in a quiescent state (p27 was highly expressed in all nuclei), in contrast to the state of irreversible proliferation arrest in senescent cells (Figure 3e,f).

We next quantitated the DP‐specific SASP factors from the conditioned medium of human DP cultures before and after senolytic treatment using protein arrays (Figure 3g). We found that the overall levels of DP‐specific SASP factors were significantly decreased in the conditioned medium after senolytic treatment (Figure 3h). Protein quantitation showed a marked decrease of p21 with IL‐6 and p‐Stat3 in senolytic‐depleted P3 cultures compared to control P3 cultures, as well as sustained expression of Gli1 and Lef1 in senolytic‐depleted P3 cultures compared to control P3 cultures (Figure 3i). We examined the SASP‐mediated interactions between senescent DP cells and nearby non‐senescent DP cells before and after senolytic treatment. We observed that the activation of IL‐6 signaling and TGF‐β signaling in non‐senescent DP cells was reversed after senolytic treatment, which subsequently recovered Gli1 and nuclear β‐catenin expression (Figure 3j,k). Moreover, the percentage of Lef1^+^ cells in cultured DP cells was significantly increased in senolytic‐depleted P3 culture compared to control P3 culture (Figure 3l,m).

**FIGURE 4:**
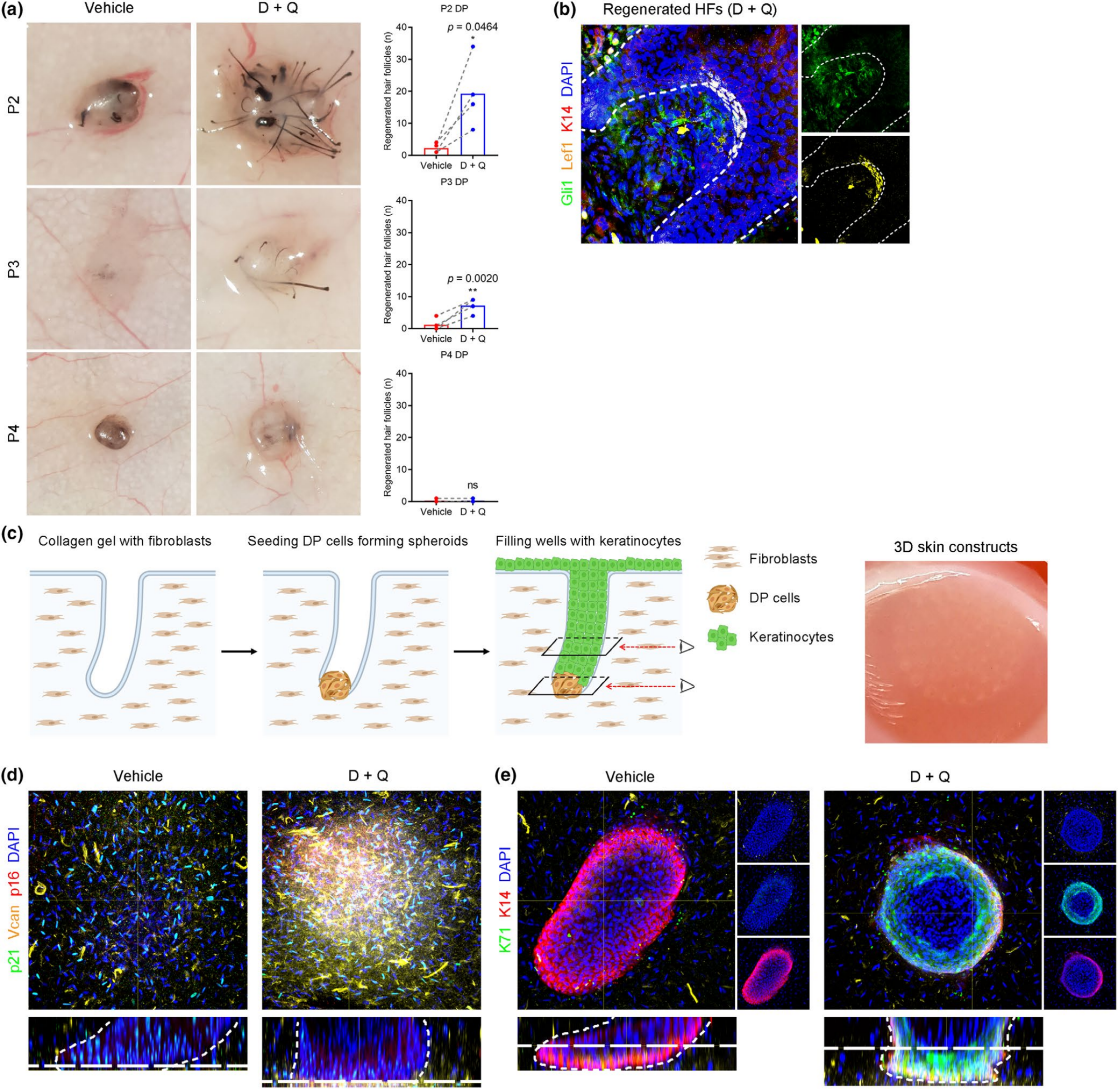
Senolytic depletion improves hair inductivity of human DP culture. (a) Patch assay and quantification (*n* = 4 [P2], 5 [P3], 4 [P4] biological replicates) showing that senolytic‐depleted DP cells showed their hair inductivity at early passage (P2), but not as significantly at later passage (P4), showing an inverse correlation between the recovery potential of hair inductivity and culture passage. (b) Immunofluorescence showing that Gli1^+^ and Lef1^+^ mesenchymal structures were observed in the proximal region of regenerated HFs in senolytic‐depleted DP group, whereas no DP structures or aggregates were detected in control DP group. (c) Experimental scheme and gross morphology of 3D human skin constructs by seeding 1 million human DP cells with and without senolytic treatment, together with 2 million human keratinocytes. (d) Whole mount immunofluorescence showing that versican^+^ DP aggregates were observed in the bottom part of microwells in senolytic‐depleted DP group, whereas no DP aggregates or structures were detected in control DP group. (e) Whole mount immunofluorescence showing that distinct HF epithelial layers including the K71^+^ inner root sheath layer and the K14^+^ outer root sheath layer were observed in senolytic‐depleted DP group, but not in control DP group. Data are presented as the mean ± s.e.m. *p < 0.05, **p < 0.01, (paired *t* test).

To investigate the functional consequencesof senolytic depletion of human DP cultures in vivo, we tested the hair inductive potential of culture DP cells before and after senolytic treatment at different passages (P2, P3, and P4) using the patch assay. Briefly, 1 day after senolytic depletion in P2, P3, and P4 DP cells cultures, we generated approximately 500 DP spheroids that contained 2000 human DP cells each (Higgins et al., 2013). The spheroids were mixed with 1 million neonatal mouse keratinocytes and injected into the back skin of SCID hairless mice. After 3 weeks, we harvested the back skin to quantitate the number of regenerated HFs and found that senolytic‐depleted DP cells showed their strongest hair inductivity at early passage (P2), compared to later passages (P3 and P4), showing an inverse correlation between the recovery potential of hair inductivity and time in culture (Figure 4a). Moreover, Gli1^+^ and Lef1^+^ mesenchymal structures were observed in the proximal region of regenerated HFs in the senolytic‐depleted DP group (Figure 4b), whereas no DP structures or aggregates were detected in the control DP group. Our findings suggest that senolytic treatment restored the hair inductive properties of human DP culture by removing repressive interactions from senescent cells, and increasing the Wnt‐active population in cultured DP cells.

Recently, we developed a biomimetic approach for human HF neogenesis within 3D human skin constructs by recapitulating the physiological organization of HF structures (Abaci et al., 2018). Using 3D printed molds, patterned array of microwells were made in a collagen gel containing human DFs, and human DP cells were seeded to form aggregates in the bottom of microwells, after which human keratinocytes were placed on top (Figure 4c). Next, we generated 3D human skin constructs by seeding 1 million human DP cells, either with or without senolytic pre‐treatment, together with 2 million human keratinocytes. Using whole‐mount scanning of human skin constructs, versican^+^ DP aggregates were observed in the bottom of the microwells in the senolytic‐depleted DP group, whereas no DP aggregates or structures were detected in the control DP group (Figure 4d). To determine whether the DP aggregates were associated with epithelial differentiation into hair‐specific lineages, we examined hair‐specific keratin expression. Distinct HF epithelial layers were observed in the senolytic‐depleted DP group, including the K71^+^ inner root sheath layer and the K14^+^ outer root sheath layer, but not in the control DP group (Figure 4e), which were comparable to those of Lef1‐transfected DP cells (Abaci et al., 2018).

To date, there are no successful treatments that can regenerate HFs in patients with permanent hair loss. Permanent hair loss after anti‐cancer chemotherapy or inflammatory disease is a serious problem for many patients, causing not only disfigurement and psychosocial stress, but also treatment refusal of chemotherapy due to the fear of hair loss. Many attempts have been made to utilize adult stem cells as a source of cells for human organ regeneration, but these approaches have not been successful without introducing cell fate modifications, which are accompanied by numerous challenges. These obstacles highlight that our understanding of why human adult stem cells exhibit poor regenerative properties when expanded *in vitro* is still incomplete.

Cellular senescence is a physiological process that evolved, in part, to enhance tissue remodeling through the secretion of the SASP, which facilitates immune‐mediated clearance of senescent cells. However, if this process is impaired and senescent cells are not efficiently cleared from tissues, the persistence of the SASP is associated with pathologic aging processes. Senescent cells accumulate in tissues and organs over the lifespan of an organism, leading to age‐related degenerative pathologies if senescent cells are not efficiently cleared from tissues (Munoz‐Espin & Serrano, 2014). In healthy tissues, senescent cells are normally cleared by the innate immune system, such as macrophages or natural killer cells (Lujambio, 2016; Munoz‐Espin et al., 2013; Pereira et al., 2019), however, this system is not completely effective and over time the progressive accumulation of senescent cells impairs the immune system leading to the accumulation of ineffective immune cells, such as macrophages, which also become senescent (Prata et al., 2018). It is also important to consider that these immune cells normally have only limited access to immune‐privileged tissues such as the central nervous system, the anterior chamber of the eye, as well as the HF (Ito et al., 2008; Walton et al., 2020).

One of the main characteristics of senescent cells is apoptosis resistance (Hu et al., 2022), thus it is likely that senescent DP survive the generalized apoptosis that depletes most DP during catagen. Also, it was recently demonstrated that follicular dermal stem cells, which normally regenerate the lower portion of the DP in physiological hair cycling, with aging lose self‐renewal, are less capable of differentiating to DP, and start exhibiting senescent features already at 12 months of age in mice (Shin et al., 2020). Therefore, in such settings, senescent cells and their secretion of the SASP may induce secondary senescence, which is defined as the induction of senescence in non‐senescent neighboring senescent cells through SASP factors. Notably, the genes encoding for the six SASP proteins identified in our microarray experiments were recently described, among many others, within the SenMayo senescence gene signature (Saul et al., 2022). IL‐6 and CCL2 are commonly described SASPs in senescence literature, including skin specific studies (Coppe et al., 2008; Jo et al., 2020; Kong et al., 2023; Saul et al., 2022; Zhang et al., 2019). Although IL‐6 is constitutively expressed by DPs, consistent with our findings, it was previously reported that balding DP cells secrete higher levels of IL‐6 compared with the non‐balding DP cells and that it inhibits hair shaft elongation and proliferation of IL‐6R expressing matrix cells in cultured HFs (Kwack et al., 2012).

It was recently reported that osteopontin secreted by senescent melanocytes could be hair‐inductive, seemingly in contrast with our conclusions (Wang et al., 2023). Although these authors demonstrated that osteopontin hyperactivates epithelial HF cells proliferation through the receptor CD44, and their experimental setting involves the constitutive presence of hair inductive DP cells. Here, we report that the accumulation of senescent DP cells secreting SASPs in the culture medium, including osteopontin, increases proportionally with passaging. We postulate that this SASP combination acts on the DP and dampens their hair inductive capacity. It is possible that the decreased hair inductivity associated with the SASPs secreted by senescent DP in vivo might be partially counterbalanced by the ability of HF epithelial cells to respond to certain SASPs secreted by other senescent cells, such as osteopontin secreted in high amounts by melanocytes, via increasing their proliferation or other mechanisms associated with hair growth.

To understand the effects of SASPs, it is imortant to know the context of the evidence emerging from the field of cellular senescence, which at times may be in contrast with evidence deriving from other scientific fields such as wound healing and immunology. It is essential to keep in mind that is the chronic and simultaneous exposure to SASPs that makes cells senescent and has detrimental effects on the microenvironment, and that these same proteins ‐ when individually studied in the context of homeostasis or acute events ‐ often produce alternative outcomes. For instance, upon tissue injury IL‐6 plays an important role in recruiting and activating immune cells and organizing tissue remodeling, but chronic IL‐6 signaling is associated with tissue fibrosis (Tanaka et al., 2014). In light of our data, it is likely that the SASPs secreted by senescent DPs act in a paracrine manner (Acosta et al., 2013) on the neighboring non‐senescent DPs leading to loss of hair inductivity, and that some of these SASPs may have different effects on other cell types within the HF, such as osteopontin, which induces the hyperproliferation of HF epithelial cells (Wang et al., 2023).

Senolytics are a novel class drugs that were developed based on the promising therapeutic potential to reverse the pathology from accumulated senescent cells and SASP. Senescent cells frequently upregulate negative modulators of apoptosis, which confer resistance to apoptosis‐inducing signals. Thus, senolytics enable senescent cells to initiate apoptosis by overcoming their resistance to apoptosis. Recently, elimination of senescent cells from the epidermis via inhibition of BCL‐W and BCL‐XL led to increased proliferation of epithelial HF stem cells (Yosef et al., 2016). Here, we showed that removal of senescent cells from human DP culture significantly improved hair inductive properties of DP cells, through reversing the repressive interactions between senescent cells and nearby non‐senescent cells, and restored the Wnt‐active population in cultured DP cells.

These findings overcome one of the major challenges in HF neogenesis, specifically, the loss of inductive DP cells after expansion in *in vitro* culture. We showed that the depletion of senescent cells using senolytic treatment effectively eliminates the SASP‐driven repressive effects originating from senescent DP cells, and potentially reactivates the inductive potential of human DP cells cultures and improves HF neogenesis in vivo.

## 1 ONLINE METHOD DETAILS

### 1.1 Ethics statement

This study was approved by the Institutional Review Board of the Columbia University (approval number: AAAI0706). All human protocols conformed to the ethical principles of the Declaration of Helsinki, and written informed consent was obtained from all human subjects. All animal procedures were performed according to the institutional guidelines and protocols as approved by the Institutional Animal Care and Use Committee (IACUC) of Columbia University Medical Center (approval number: AC‐AABG4550).

### 1.2 Human primary cell cultures

For the isolation of human DP cells, the intact DPs were dissected from individual HFs that were morphologically determined to be in the anagen stage. Human intact DPs were placed on culture plates and submerged in Dulbecco's modified Eagle's medium (DMEM, Thermo Fisher) with 20% fetal bovine serum (FBS) (certified, United States #16000044 Thermo Fisher) and 1× penicillin–streptomycin (Gibco) at 37 °C in a 5% CO_2_ atmosphere. The same batch of FBS was used throughout the experiments. FBS was thawed overnight at 4°C, heat inactivated by incubating at 56°C for 30 min, aliquoted, and stored frozen at −40°C. On the 5th day of culture, the medium was changed to DMEM with 10% FBS and 1× penicillin–streptomycin at 37 °C in a 5% CO_2_ atmosphere. The culture medium was changed every 2 days. For routine passaging, cells were subcultured at 70%–80% confluence using 0.25% trypsin solution (Thermo Fisher).

### 1.3 Immunofluorescence staining

For immunofluorescence on tissue sections, mouse back skins were harvested then embedded and fresh frozen in OCT (Thermo Fisher). Sections were cut at 8‐μm thickness with a cryotome (Leica). After drying, sections were fixed to slides with 4% paraformaldehyde/PBS for 10 min at 25°C. After washing in PBS, sections were then permeabilized in 0.3% Triton X100/PBS for 15 min at 25°C before blocking in 0.5% normal donkey serum (Jackson ImmunoResearch) for 1 h at 25°C. Primary antibody labeling against Ki67 (1:250), p16 INK4A (1:250), p21 Waf1/Cip1 (1:400), p27 Kip1 (1:1600), IL‐6 (1:1000), p‐Stat3 (1:100), t‐Stat3 (1:1000), Gli1 (1:200), Lef1 (1:200), Gata4 (1:200), β‐catenin (1:200), and versican (1:200) was performed overnight at 4°C. Secondary antibody labeling was done with donkey anti‐rabbit, rat, guinea pig, chicken, or mouse antibodies conjugated with AlexaFluor 488, 594, or 647 (Invitrogen) for 1 h at 25°C. Nuclei were labeled with DAPI (Thermo Fisher), and stained sections were mounted in ProLong Gold Antifade Mountant (Invitrogen). Antibodies used in this study are listed in the Table S1.

### 1.4 Whole‐mount immunofluorescence staining

For whole‐mount immunofluorescence on pieces of intact tissues, mouse back skins were harvested, and cut into 1 × 0.8 cm strips fixed in 4% paraformaldehyde/PBS overnight at 4°C before peeling away the panniculus carnosus muscle. Subsequent steps were then performed in a 24‐well plate. Skins were then treated with a 0.3% Triton X‐100, 5% donkey serum, 20% DMSO/PBS solution for 8 h before primary antibody labeling against p16 INK4A (1:250), p21 Waf1/Cip1 (1:400), versican (1:200), Krt71 (1:100), and Krt14 (1:500) in the Triton X‐100/donkey serum/DMSO/PBS solution for 5 days at 25°C with gentle shaking. After washing in 0.3% Triton X‐100/PBS solution for 8 h at 25°C with buffer changes every 30 min, tissues were then stained with donkey anti‐rabbit, rat, guinea pig, chicken, or mouse antibodies conjugated with AlexaFluor 488, 594, or 647 (Invitrogen) in the Triton X‐100/donkey serum/DMSO/PBS solution used similarly with primary antibody labeling for 3 days at 25°C with gentle shaking. Finally, tissues were washed again in 0.3% Triton X‐100/PBS solution for 8 h at 25°C with buffer changes every 30 min and counter stained with DAPI. Optical clearing was then performed by dehydrating tissue in 50:50 methanol/water for 5 min then a series of three 100% methanol treatments for 30 min at 25°C. Final clearing was performed in a BABB (benzyl alcohol/benzyl benzoate, 1:2 ratio, Sigma) solution until visibly clear then mounted in residual BABB held in a custom chambered glass slide. Antibodies used in this study are listed in the Table S1.

### 1.5 Imaging and microscopy

Immunofluorescence‐stained sections and whole‐mount tissues were imaged with a Leica Dmi8 with Stellaris 5 confocal microscope system equipped with Leica LAS X software. Image stacks were acquired with a x–y resolution of 0.284 μm and a z‐step of 1 μm, and post‐processed and adjusted for brightness and contrast, and reconstructed into 3D objects using ImageJ/FIJI (NIH). The hair bulb diameter of regressing HFs, the number of apoptotic epithelial cells, the number of dermal immune cells were measured using the 3D objects reconstructed from whole‐mount immunofluorescence‐stained tissues using ImageJ/FIJI.

### 1.6 Human cytokine and chemokine array

A protein array system was utilized to determine the presence or absence of a total of 80 cytokines and chemokines in the conditioned medium of human DP culture. Human Cytokine Array Membrane Kits (AAH‐CYT‐5, RayBiotech) were used according to the manufacturer's instructions. The signal was quantified using ImageJ. Semi‐quantitative positive results were determined based on the presence of signals in the location for the protein of interest and normalized during the analysis of the raw data, which was performed using RayBiotech Microsoft® Excel‐based Analysis Tools, by selecting a reference array (the culture medium in our case) as recommended by the manufacturer.

### 1.7 Western blot analysis

Total protein from human DP cells was extracted using RIPA lysis buffer (Millipore) according to the manufacturer's instructions. Equal amounts of total protein were separated by electrophoresis using 8%–10% sodium dodecyl sulfate‐polyacrylamide gels and transferred onto polyvinylidene fluoride membranes (Amersham). The blotted membranes were cut horizontally according to the size marker and incubated with the following primary antibodies corresponding to the target protein size at 4 °C overnight: p21 Waf1/Cip1 (1:1000), IL‐6 (1:1000), p‐Stat3 (1:1000), t‐Stat3 (1:1000), Gli1 (1:1000), Lef1 (1:1000), and β‐actin (1:5000). The membranes were probed with anti‐rabbit IgG and anti‐mouse horseradish peroxidase‐conjugated IgG antibodies (Thermo Fisher) at 25 °C for 1 h. The blotted membranes were restored by removing bound primary and secondary antibodies using Restore™ PLUS Western Blot Stripping Buffer (Thermo Fisher) and reprobed for the different size target protein compared to the original target protein. Antibody–antigen complexes were visualized using an enhanced chemiluminescence system (Thermo Scientific) and captured by an ChemiDoc™ Imaging System (BioRad).

### 1.8 Generation of 3D human skin constructs

All 3D‐printed molds were designed and drawn using the computer‐aided design (CAD) software, Solidworks. Each HF‐like extension on the molds was 500 μm in diameter and 4 mm in length. The molds with hair densities (81 HFs/cm^2^) were 3D‐printed using Objet24 3D‐Printer (Stratasys) which uses a UV‐curing material VeroWhite (Stratasys). 3D skin constructs were generated in six‐well‐plate transwell inserts similar to the method described previously^12^. The dermal compartment was prepared by adding 4 mL of type I collagen matrix containing 1.25 × 10^5^ fibroblasts per mL into the transwell inserts and polymerized around the 3D‐printed HF molds placed on top of the gel at 25°C for 30 min. After complete polymerization, the molds were removed and 100 μL of human DP cell suspension (total 1 × 10^6^ cells) was added on top of the gel. The constructs were cultured overnight in DMEM with 10% FBS for aggregate formation, after which human keratinocytes (total 2 × 10^6^ cells) were added on top of the gel. The constructs were maintained in low calcium epidermalization medium submerged for 2 weeks.

### 1.9 Patch assay for HF reconstitution

Human DP 3D spheroids were formed with cultured DP cells at different passages by using AggreWell plate (#34411, Stemcell Technologies) containing of 2000 cells per each microwell in DMEM with 10% FBS at 37 °C in a 5% CO_2_ atmosphere for 48 hours. Human DP 3D spheroids (total 1 × 10^6^ cells) were combined with freshly isolated mouse neonatal epidermal cells (total 1 × 10^6^ cells) and transplanted subcutaneously into the backskin of 8‐week‐old severe combined immunodeficiency hairless outbred mice (SHO mice; Jackson Laboratory). After 4 weeks, the host mice were sacrificed to harvest regenerated tissues.

### 1.10 Global gene expression analysis

The global gene expression data were imported from our datasets (accession GEO GSE44765). Differentially expressed gene analysis was performed using the software R with the DESeq2 package (adjusted *p* < 0.05), and the results were visualized by using the EnhancedVolcano package. For the hierarchical clustering, a mean expression matrix was calculated by averaging the gene expression values of all three replicates. Then, the gene expression matrix was log_2_ transformed and normalized among the groups, and the heatmap was subsequently generated using the pheatmap package. Based on the cluster list of upregulated genes over passage, biological pathway analyses were performed using the gProfileR package.

## 2 QUANTIFICATION AND STATISTICAL ANALYSIS

### 2.1 Statistics and reproducibility

No statistical methods were used to predetermine sample size. The experiments were not randomized, and investigators were not blinded to allocation during experiments and outcome assessment. Number of biological replications is specified in the figures and figure legends. Data are presented as mean ± s.e.m., with biological replicates on the background of graph. Two‐tailed unpaired Student's *t*‐test, two‐tailed paired Student's *t*‐test, Welch's *t*‐test, linear regression test, two‐way analysis of variance (ANOVA) with Sidak's multiple comparisons test, two‐way analysis of variance with Dunnett's multiple comparisons test, or repeated measure one‐way analysis of variance with Sidak's multiple comparisons test were used to analyze data sets with two or more than three groups. Generation of data plots and statistical analyses were performed using Excel (Microsoft) and Prism (GraphPad). *p* < 0.05 were designated as significant and symbolized as *p < 0.05, **p < 0.01, ***p < 0.001, ****p < 0.0001 with precise *p*‐values supplied in the figures and figure legends.

## AUTHOR CONTRIBUTIONS

J.Y.K. executed the experiments, analyzed the data, designed the figures. J.Y.K. and A.P. drafted the manuscript and figures. H.E.A. and A.M.C. supervised the data analysis and contributed to the manuscript. All authors read and approved the final manuscript.

## FUNDING INFORMATION

This project was supported in part by private philanthropic funding. Ongoing work on cellular senescence in the skin more broadly was supported in part by the National Cancer Institute through our participation in the Cellular Senescence Network (SenNet) from the National Institutes of Health.

## CONFLICT OF INTEREST STATEMENT

The authors declare no conflict of interest. Columbia University has filed patents on the use of human DP cells in HF neogenesis.

## Supporting information


Figure S1.

Table S1.

## Data Availability

The accession numbers for the global gene expression profiling reported in this paper are available in the NCBI Gene Expression Omnibus (GEO) archive (accession GEO GSE44765). The source data underlying figures are provided as a Source Data file. All Supplemental materials for this article are available in the Supplemental Information file. The routine scripts used for data analysis in this study are available upon reasonable request.
