# Supplementary material for: Restoration of hair follicle inductive properties by depletion of senescent cells
Source: Aging Cell. 2024 Nov 29;24(1):e14353. doi: 10.1111/acel.14353 (PMC11709086; doi:10.1111/acel.14353)
Supplement: Supplementary file 1 — Figure S1. [file ACEL-24-e14353-s002.docx]

**Restoration of hair follicle inductive properties by depletion of senescent cells**

Alberto Pappalardo^1^, Jin Yong Kim^1^, Hasan Erbil Abaci^1^, Angela M. Christiano^1,2,^*

**Supporting Information**

Supplementary Figures 1-2


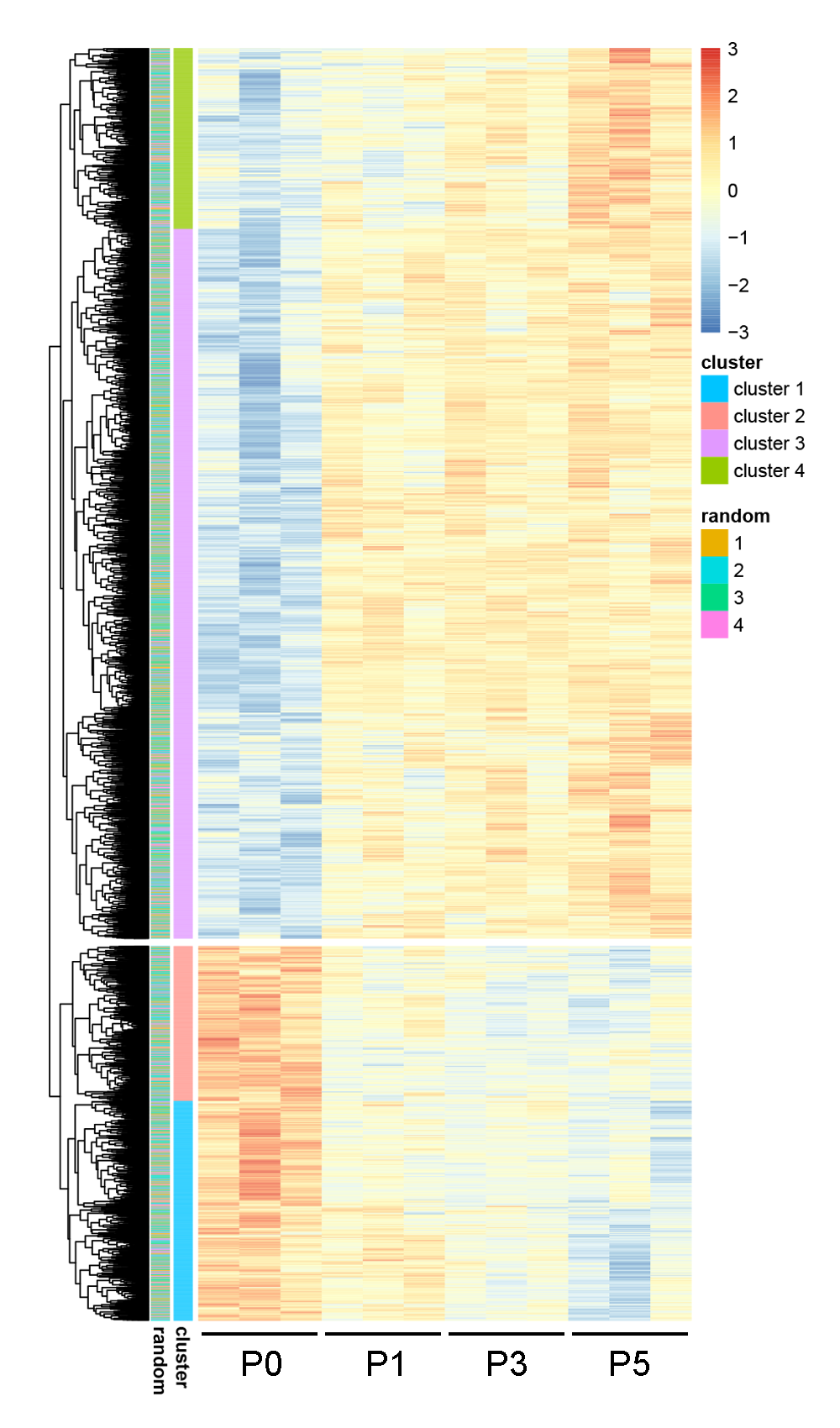


**Supplementary Figure 1.** **Global gene expression profiling on human DP culture**

Heatmap of differential gene expression showing that 3738 genes (16%) were upregulated while 1444 genes (6.3%) were downregulated over passage in human DP culture (*n* = 3 biological replicates).


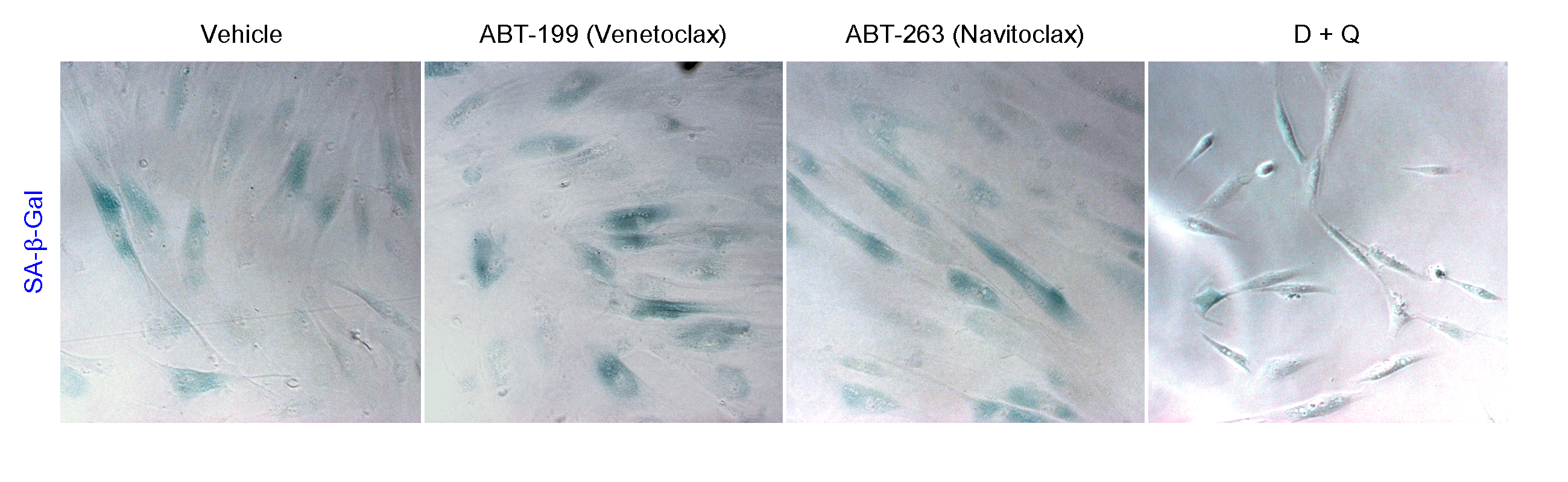


**Supplementary Figure 2.** Identification of the most potent senolytic drugs for human DP culture

SA-β-Gal activity of human DP culture after senolytic treatment including ABT-263 (Navitoclax), ABT-737, ABT-199 (Venetoclax), and dasatinib plus quercetin (D + Q). Note that D + Q was the most potent for depleting senescent cells from human DP culture.
